# Bringing disgust in through the backdoor in healthy food promotion: a phenomenological perspective

**DOI:** 10.1007/s11019-021-10037-0

**Published:** 2021-06-28

**Authors:** Bas de Boer, Mailin Lemke

**Affiliations:** 1 University of Twente, Enschede, The Netherlands; 2 Delft University of Technology, Delft, The Netherlands

**Keywords:** Obesity, Disgust, Healthy food promotion, Phenomenology

## Abstract

Obesity has been pointed out as one of the main current health risks leading to calls for a so-called “war on obesity”. As we show in this paper, activities that attempt to counter obesity by persuading people to adjust a specific behavior often employ a pedagogy of regret and disgust. Nowadays, however, public healthcare campaigns that aim to tackle obesity have often replaced or augmented the explicit negative depictions of obesity and/or excessive food intake with the positive promotion of healthy food items. In this paper, we draw on a phenomenological perspective on disgust to highlight that food-related disgust is connected to the character and behavior of a perceived individual even in the context of promoting healthy food items. We argue that the focus on “making the healthy food choice the easy choice” might be an important step towards the de-stigmatization of people that are affected by obesity. However, so we suggest, this focus threatens to bring back an image of individuals affected by obesity as disgusting “through the backdoor”. It does so not by portraying bodies with overweight as disgusting, but instead by implying that lifestyle choices, character and habits of people that are affected by obesity are markers of a lack of control. We argue that the close relationship between disgust and the perception of self-control in the context of obesity should be taken into consideration in the context of assessing the implications of new health promotion strategies to minimize the risk of stigmatizing people.

## Introduction

Obesity has been pointed out as one of the main current health risks leading to calls for a so-called “war on obesity”.^1^ There is much debate about the effects of obesity on individuals and society, as well as on how public health campaigns can be used to decrease the amount of individuals affected by obesity. In the past, a common approach has been to depict people who are affected by obesity as “disgust eliciting” (Lupton 2013, 2015) explaining their increased body weight in terms of too much food intake and too little exercise (Gard and Wright 2005). Typically, public health campaigns involved graphic imagery of the alleged ugliness of bodies with overweight, the greasiness of fast-food products, and the way those products contaminate internal organs (Lupton 2015, 5). This focus on assigning the responsibility of obesity solely to the individual has been criticized as leading to the stigmatization of people affected by overweight (Shugart 2013; Ulijaszek and McLennan 2016), eventually leading to a situation in which these people are less likely to try to find appropriate care (Phelan et al. 2015). This is one of the reasons that the recent emphasis in efforts to counter obesity seems to have shifted to making “healthy food choices the easy choice” (e.g., LiveLighter 2021a). In this paper, we argue that such approaches often bring back an image of individuals affected by obesity as disgusting “through the backdoor”, not by portraying their bodies as disgusting, but instead implying that their lifestyle choices, character and habits are potentially disgust eliciting, and hence leads to stigmatization.

Our argument is grounded in a narrative literature review of the relationship between disgust, food and obesity. We included publications from the fields of psychology, philosophy, health research, sociology, design and cultural history. Although it is sometimes claimed that obesity is a relatively recent phenomenon, there is a longstanding conversation around fat and its implications that can be traced back to historical and religious texts (Haslam 2007; Haslam and Haslam 2009; Stunkard et al. 1998). While medical findings strongly influence our perception of obesity, there are also underlying moral standards and norms at play that shape how obesity is viewed within society (Patterson and Johnston 2012). In this article, we intend to highlight the complex role of disgust in the context of healthy food promotion. The purpose of doing so is to illustrate the different ways in which forms of health promotion can forge a connection between food and disgust, leading to the (unjustifiable) stigmatization of individuals affected by being overweight.

This paper is structured as follows: First, we summarize the current discourse and research findings around obesity and its relation to self-control. Second, we present a model that outlines the variety of ways in which disgust can be connected to food. Third, we reflect on some central approaches in health promotion, showing how they (implicitly) employ disgust as a tactic for establishing behavior change. In a consequent step, we explain how disgust is embedded in healthy food promotion from a phenomenological perspective. Building on this perspective, we subsequently show how particular entities are experienced as disgusting due to certain performative work, and point to the close connection between the experience of disgust and one’s personal identity. In conclusion, we suggest that health promotion strategies could benefit from recognizing the complex ways in which food and disgust are connected.

## Obesity, stigmatization, and self-control

The current debate around obesity includes numerous research fields with often conflicting views. Obesity is defined as a medical condition, sometimes even a disease in its own right, signified by abnormal or excessive levels of adipose or fatty tissue (World Health Organization 2000). However, having an increased body weight can also be seen as part of one's social identity influenced by social, cultural and historical norms (Patterson and Johnston 2012). Obesity is commonly measured with the body mass index (BMI) and classifies people as being "overweight" with a BMI greater than or equal to 25 and "obese" with a BMI greater than or equal to 30 (World Health Organization 2021). In this paper, we do not differentiate between people with overweight and people with obesity and the stigma they experience, since the tools and categories which are used to define these groups, have changed over the years and have been pointed out to carry little meaning in the context of weight-related stigma (Gard and Wright 2005; Puhl and Brownell 2003). For example, the cut-off points of the BMI can vary between countries and often do not consider variations in human body sizes (e.g. size, amount of fat, muscles and bones) (Gard and Wright 2005; James 2008).

The literature investigating and discussing the development and impact of obesity is vast. However, the field can be usefully divided into two main research silos, sometimes referred to as "alarmists" on the one side and "sceptics" on the other (e.g., Penney and Kirk 2015). The silo that sees obesity as a worldwide evolving disease points out that it poses a serious health threat to individuals and a financial burden to society in general. The other silo argues that obesity is a construct influenced by social norms and perceptions, and positioning it as a disease contributes to moral panic, and unnecessarily negative conceptions of fat (e.g., Gard and Wright 2005; Gard 2011; Patterson and Johnston 2012). The public view of obesity seems to be dominantly influenced by the “alarmist” silo. Furthermore, medical knowledge of obesity is often simplified in the public discourse, and tends to neglect the complex picture that medical research paints of the condition. For example, an increased BMI is commonly correlated with a reduction of health. However, it seems that the relative risk of dying plotted against BMI seems to follow a U shape where the greatest risk of dying is both for the very lean (BMI under 21) and the very obese (BMI great than 31) (Waaler 1987).

Furthermore, the dominant public perspective on obesity seems to be based on a mechanistic understanding of the body as a machine that converts food into kinetic or thermal energy required for the body to function. Following the machine analogy, increased body weight is explained by the fact that either too much food is consumed or too little energy is used. This perspective poses the responsibility and capability to manage weight primarily on the individual and neglects factors such as time, gender and culture (Gard and Wright 2005). The public sphere has taken up this dominant message seeing people with increased weight as sick and responsible for their condition, and urge them take up this responsibility by lowering the consumption of allegedly unhealthy food items (Patterson and Johnston 2012).

Addressing the “obesity epidemic”^2^ in terms of personal responsibility turns obesity into a moral concern. This development mimics negative historical conceptions of fat as the result of irresponsible behavior.^3^ The ancient Greeks promoted moderation and avoidance of food excess based on the individual’s responsibility to feel and be healthy. In medieval times, fat was in Europe and Asia related to the deadly sins of gluttony and sloth, leading to a clear negative conception of fat (Haslam and Haslam 2009; Stunkard et al. 1998). Around this period being fat starts being framed as a moral failing which is still visible in the current public discourse around obesity (Farrell et al. 2016). However, gluttony was not necessarily connected to being overweight but also by eating too much food and too many delicacies. Following this logic, a glutton could also be a lean person (Haslam and Haslam 2009). The late eighteenth century was a tipping point in the public perception of fat, marking the establishment of a widely shared anti-fat attitude reinforced through medicine, as well as through aesthetic and moral ideals. Obesity is increasingly associated with multiple uncivilized traits, including being sweaty, immoral, and weak. Having a lean body allows distinguishing oneself from “the others” even in a broader cultural and geographic context (Forth 2012a, b). Signs of increased weight were and still are interpreted as signs that an individual has “lost control” and the body is interpreted as an indicator of one’s personality often leading to the stigmatization of the individual in question (DeJong 1980; Pausé 2017). And the perception of obesity as a disease goes so far that people interpret obesity as a contagious sickness cue similar to influenza (Tapp et al. 2020).

This idea of personal responsibility has been central in the public discourse around food intake (Brownell et al. 2010), and indulgence is typically interpreted as deviant behavior (Inthorn and Boyce 2010). Studies indicate that nations with a strong individualistic perspective founded on the idea that "people get what they deserve" show higher anti-fat attitudes and prejudices. In these nations, obesity prejudice correlates with the perception of it being based on willpower and self-control (Crandall and Biernat 1990; Crandall et al. 2001).

On the basis of the above mentioned studies, it can be argued that obesity is stigmatized, such that being overweight is treated as a personal attribute which is “deeply discrediting” (Goffman 1963, 3), and violates established norms of what is considered “normal”. Although a comprehensive theory of weight-related stigma is still missing (Puhl and Brownell 2003), it is notable that people affected by obesity are routinely depicted in a negative manner within education, health care provision, interpersonal relationships, media and advertisement (Puhl and Heuer 2009). Furthermore, many anti-obesity campaigns are premised on the idea that people should be in control of their own weight and that stigma will motivate them to engage in “appropriate” behavior (Vartanian and Smyth 2013). This creates an atmosphere of fear and disgust in the hope of future health benefits and reinforces a control and blame approach to obesity (Couch et al. 2018), which constitutes a situation in which it seems that there is an expectation that people affected by obesity should support particular health norms, as well as should attempt to realize it (cf. Goffman 1963, 17). Every failure to realize these norms, then, can be treated as a failure on the part of the individual affect by being overweight, such that people with an increased body weight start to internalize that they indeed lack self-control and can be rightfully blamed (Goffman 1963, 19; Williams and Annandale 2020). This in turn can cause additional issues, including maladaptive eating behavior, avoidance of exercise, or being unwilling (or too ashamed) to seek appropriate care (Puhl and Heuer 2009; Williams and Annandale 2020). We will focus on how the use of disgust in the context of health promotion contributes to this process in the next two sections. First, we propose a model of how being overweight can elicit disgust (section ‘A model of how being overweight can elicit disgust’), and subsequently we show how this model is (implicitly) employed in healthcare campaigns.

## A model of how being overweight can elicit disgust

**Fig. 1:**
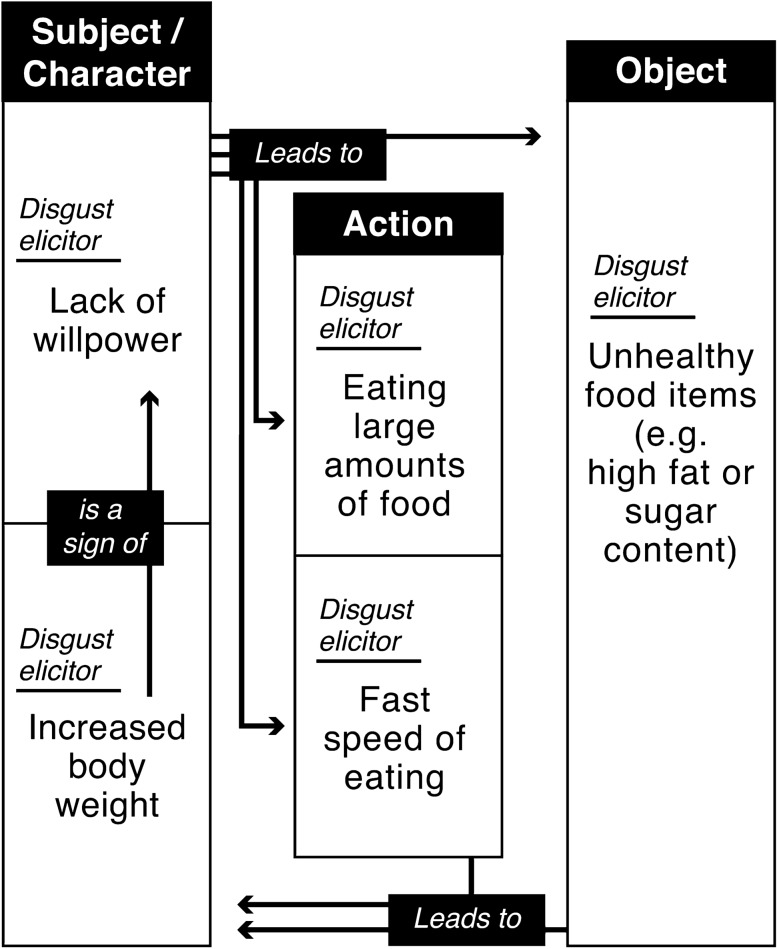
A model of how being overweight can elicit disgust. The model shows the relationship between the subject (the person), action (the act of eating) and the object (the food item). All of these elements are potential disgust elicitors

In Fig. 1, the different ways in which obesity can elicit disgust are schematically represented. We take as a starting-point a widely accepted health identity that has a long history in Western countries in which having a certain body weight—nowadays often conceptualized in terms of having a “normal” BMI—is taken to positively correlate with being healthy (Gard and Wright 2005). We do so because advertisements for healthy food as well as public healthcare campaigns more generally seem to be (implicitly) premised on this image (Lupton 2015). Departing from this particular image, being affected by overweight is perceived as resulting from a connection between the qualities of a subject (e.g., its body or character); the actions that the subject performs (e.g., eating a certain amount of food at a particular pace); and the food items that the subject consumes (e.g., consuming particular food items) (see Fig. 1 below). A sign of being healthy, then, is to be a subject with a particular body and certain character traits (e.g., self-control, moderation) allowing for this particular size, such that the subject consumes an *appropriate amount* of food in an *appropriate way*, as well as the *appropriate food items*.

As will be shown below, healthcare campaigns often use the above-mentioned accepted health image in attempts to counter the rise of obesity by presenting deviations from a healthy pattern in one or more of the three domains (subject, action, object) as disgust eliciting. They might construct either the size of one’s body, one’s character traits, the type of food one consumes, how the food is consumed, or the connection between these elements as disgust eliciting. Each of these elements can be considered as disgust eliciting, as they are part of a logic in which the qualities of objects, subjects, and actions are closely connected. As we will elaborate on below, explicitly portraying these elements as disgust eliciting gives rise to the stigmatization of individuals affected by obesity.

The promotion of a healthy lifestyle including a diet of particular food items that are allegedly healthy is often presented as a more positive and less stigmatizing way to fight obesity. Instead of focusing on the subject and the action it performs, it actively promotes the consumption of certain objects (food items) as having a positive effect on one’s health and wellbeing. This attempt to isolate food items and eliminate their potential disgust eliciting character, presupposes that it is altogether possible to view them independently of the subject that consumes them in the act of eating. However, as our model suggests, and as will be clarified throughout the paper, the subtle ways in which entities elicit disgust makes impossible the neat isolation of food items from the subject that consumes them in a certain way. When this is not sufficiently attended to, an image of obesity as resulting from lacking certain character traits such as responsibility and self-control is brought back in through the backdoor, thereby potentially reinforcing the stigmatization of and weight bias towards individuals who are affected by obesity.

## Public campaigns and the “war against obesity”

Current public health campaigns often appeal to (negative) emotions to motivate people to engage in allegedly healthier behaviors (Wakefield et al. 2010; Lupton 2015). Also, much research currently focuses on how healthy behavior change can be established through explicitly targeting emotions such as fear and disgust as a way of preventing people from consuming certain products (Morales and Fitzsimons 2007; Tannenbaum et al. 2015; Collymore and McDermott 2016; Clayton et al. 2017). For example, graphic images of the effects of tobacco use on one’s body are frequently used to communicate the negative effects of habitual smoking, and have been found to be effective in increasing smoking cessation rates (Clayton et al. 2017).

In the context of anti-obesity campaigns a similar appeal to negative emotions is made (Puhl et al. 2013a, b; Couch et al. 2018). As we mentioned above, disgust is an often-targeted emotion in this context: it seems to be a widely shared assumption that the “war on obesity” is thought to be fought most effectively through a *pedagogy of disgust* (Lupton 2015).

Public health campaigns have used different approaches to position the “subject/action/object” (see Fig. 1) in the context of food as disgust eliciting to evoke the intended behavior change. Campaigns have used claims such as “No child dreams of becoming an overweight adult” (Latinworks 2015), referring to the visual metaphor of freeing oneself from the “imprisonment” of the physical body (Ayman 2009), or the stated fact that “Fat can’t hide” (Kolle Rebbe 2014), suggesting that the “real you” is somewhere hidden below all your weight and just needs to be freed. Furthermore, the subject is also presented as disgust eliciting in the form of “body envelope violation” by focusing on the inner organs of the person (Couch et al. 2018) and highlighting the effect of “unhealthy eating habits” by showing photos of human organs emphasizing the toxic nature of the accumulated fat that is shown on the image or video (e.g. LiveLighter 2021b). This is an approach that resembles anti-smoking advertisements of cigarette packaging displaying black smoker lungs. Furthermore, the subject is often portrayed in a de-identifying or stigmatizing and ridiculing manner as part of public campaigns and public news articles (Heuer et al. 2011; Couch et al. 2018).

Other campaigns connect the action and object level (Fig. 1) and portray eating habits and food items considered to contribute to obesity as disgust elicitors. For example, the New York City Health Department launched a campaign in 2010 claiming that drinking one can of soda a day makes you 10 lb heavier over the course of a year. The images and video were chosen to have a “major gross out factor” (Hartocollis 2010) and showed blobs of yellow fat on people’s plates or people drinking fat out of a can instead of soda. Fat in this context is either described as a toxic substance to the viewer or has slimy and yellow consistency, seemingly contaminating everything it touches. A similar approach was recently employed by the LiveLighter (2020) campaign in Australia. The “16 Teaspoons of Sugar” advertisement. The clip shows a girl seemingly drinking out of a can, but instead of liquid, there is just sugar coming out of the can. She starts looking around the family, which is gathered around the TV, and they are also eating hands full of sugar instead of food. The clip is constructed as a kind of nightmare out of which the girl wakes up in the end by grabbing a glass of water instead of soda. The disgust eliciting effect of the clip relies on exaggerating how much sugar people consume when they chose “unhealthy” food. It is noticeable that all of these campaigns mostly focus on the excessive consumption of food items that are not considered to be luxurious (such as e.g., expensive meat, high-quality wine, or exclusive vegetables such as artichokes). This suggests that one important concern of public health campaigns is to counter specific ways of advertising present in the “fast-food industry,” which can be singled out a contributor to gaining weight (e.g., Hoek and Gendall 2006).

Given that the number of children with overweight is increasing rapidly (e.g. see World Health Organization 2018), parents (mainly mothers) are often marked as potential wrongdoers to their offspring (De Brún et al. 2013) responsible for the food that their children eat, as well as the amount of physical activity that they engage in. Also, in this case, an appeal is often being made to things that people tend to find disgusting. For example, in the advertisement *Break the Habit* from the Australian *The Precinct Studios,* a mother is preparing a syringe of heroin, which is during the advertisement gradually transformed into a hamburger that the mother feeds to her child, thereby explicitly linking the consumption of junk food to the image of the “junkie” (The Precinct Studios 2010). Other advertisements have portrayed an infant at a mother’s breast that is depicted as a greasy hamburger that the infant starts eating (Paim 2015), thereby linking the unhealthy food choices of the parents to the nutritional intake of their offspring. In such cases, the (presumably wrong) food choices that parents make are extended to the bodies of their children that are negatively affected by the parents’ lack of self-control.

Such strategies have been extensively criticized for being premised on an accepted health image and the belief that one’s weight can be controlled (O’Hara and Gregg 2006; Vartanian and Smyth 2013). By presenting individuals affected by obesity as having insufficient self-control resulting in the inability to suppress their urges, the pedagogy of disgust effectively establishes a close link between being fat and having lost control. As a result, signs of obesity are interpreted to be indicators of eating too much, eating unhealthy food and being unfit; issues that need to be addressed and corrected for on the level of the individual (Lupton 2015). Such stigmatizations are found to be ineffective and have a negative effect on mental health since they have negative consequences for the self-image and self-understanding of individuals affected by obesity (Puhl and Latner 2007; Puhl and Heuer 2009; Vartanian and Smyth 2013).

Deborah Lupton's critique on public health campaigns that employ disgust primarily focuses on the position of subject/action/object as explicit disgust elicitors leading to the stigmatization of people affected by obesity (see Fig. 1). In addition, so we argue, also “positive” health promotion could also give rise to disgust and obesity stigma. In recent years there seems to be a shift to develop alternatives that do not explicitly involve disgust and stigmatization, but instead focus on the positive effects of living a healthy lifestyle that includes the consumption of healthy food items (Hansen et al. 2016). This is increasingly alluded to as a more effective and potentially less stigmatizing way of countering obesity. For example, efforts are made to change the food available in school canteens, such that children are getting used to consuming healthy food at an early age. Furthermore, attempts are being made to place emphasis on how the consumption of healthy food items has a positive effect on one’s energy levels and/or life span. The promise of such strategies is to de-stigmatize obesity and empower individuals to make healthy food choices to increase their own wellbeing, thereby departing from the presentation of individuals affected by obesity as having a diseased, contaminated (i.e., disgusting) body that they are unable to take care of.

Promoting the positive effects of healthy food consumption is an important step forward in the de-stigmatization of obesity, because it draws attention away from the individuals affected by obesity. However, it shares with the pedagogy of disgust the idea that self-control and “making the right choice” are crucial when countering obesity. Appropriate self-management remains a crucial factor in good self-care, such that individuals who make food choices that run counter to the norms set by healthy professionals can be understood to be engaging in “risky behavior” that they should abstain from (Devisch and Vanheule 2015).^4^ This mechanism is especially visible in the design of persuasive technologies that allegedly empower people to live a more healthy lifestyle; often these are explicitly designed to make people aware of their unhealthy (food-related) habits (Chatterjee and Price 2009), thereby reinforcing the connection between “healthy food” and “healthy eating” with the concept of self-control (Askegaard et al. 2014). As a result, one’s “risky” lifestyle choices and habits remain to be considered as stemming from a weakness of will that is in need of correction.^5^ In the remainder of this paper, we explain that explicitly forging a connection between being overweight and the absence of self-control remains to present people affected by obesity as potential disgust eliciting, and hence contributes to weight stigma.

## A functional perspective on disgust

Now that it is established that the primary discourse about how to fight the “war on obesity” remains one in which being overweight is a matter of insufficient self-control, we can return to the topic of disgust. Disgust has been a topic of interest since antiquity, famously described by Plato in terms of how it evokes both a response of repulsion and of attraction in *Book IV* of *The Republic* (439e-440a; Korsmeyer 2011). However, currently disgust is a widely studied topic; an interest that can be traced back to research efforts by the psychologists Rozin and Fallon (1987). In the context of this article, we refer to disgust as “*a strong feeling of dislike, finding a thing very unpleasant, or against one’s principles*” (Ehrlich 1986). This can include strong responses including loathing as well as less intense ones including dislike.

**Fig. 2:**
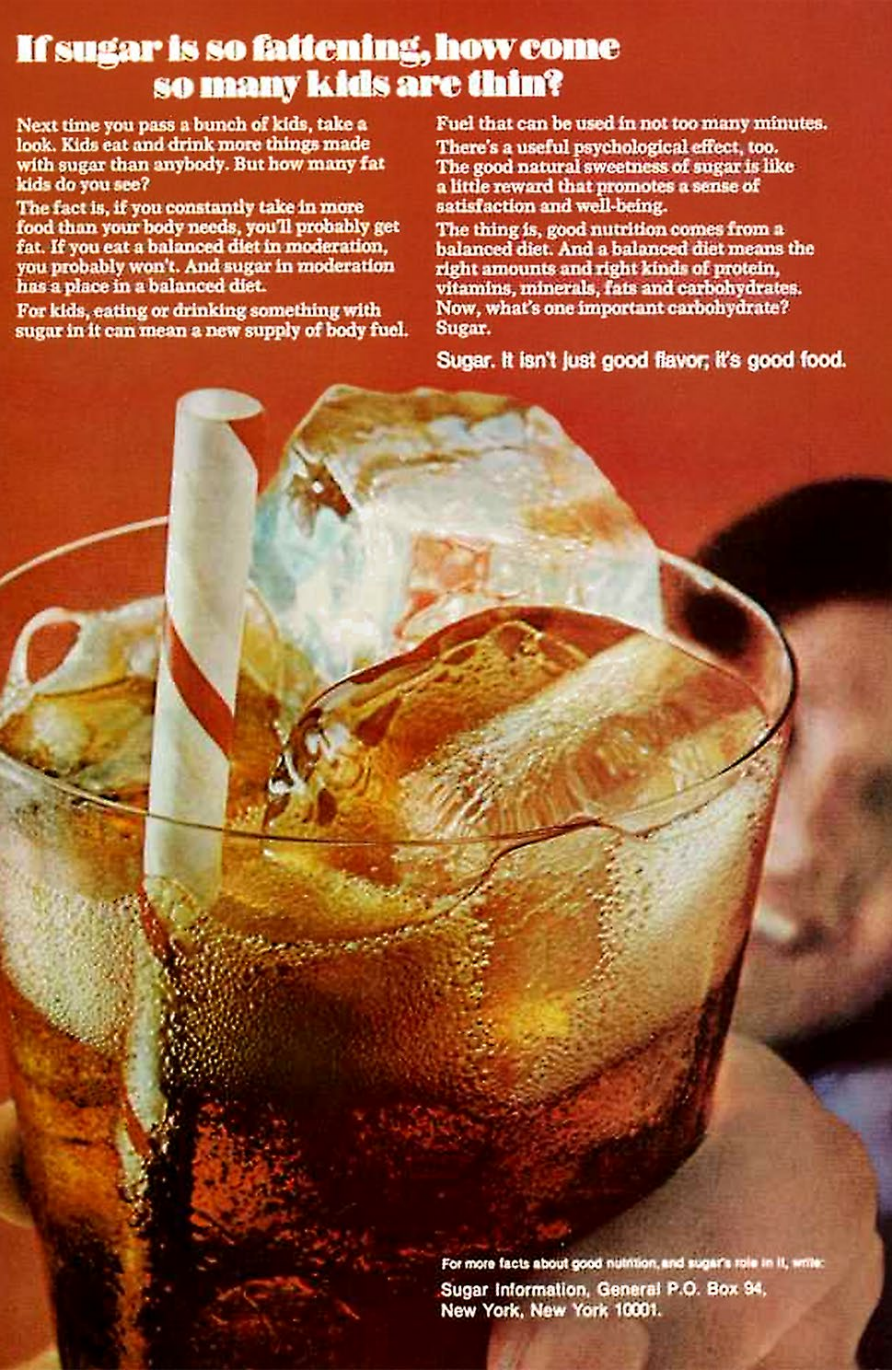
The image shows an advertising campaign from 1977 promoting the consumption of sugar as part of a healthy diet. The headline of the poster reads "If sugar is so fattening, how come so kids are thin?" followed by a copy text that describes that sugar is beneficial as part of a balanced providing fuel for the body and even has a useful psychological effect on the consumer. Photo: “Vintage delusion” by Lester (2010) is licensed under the Creative Commons license CC BY 2.0. The image contrast was adjusted to increase legibility.

There are a number of theories focusing on the different domains and functional levels of disgust that take it to be a preventive mechanism against any kind of contact with poisons and pathogens (Rozin and Fallon 1987; Miller 1998; Kelly 2013; Tybur et al. 2013). Yet, there is still a lack of an accepted definition and theory of disgust outlining its core (Strohminger 2014). We will leave the discussion about the differences and similarities of the proposed theories and frameworks to others due to our focus on the experience of disgust rather than the conceptual underpinning of the emotion. However, we will briefly focus on the *entanglement theory* by Daniel Kelly to point to disgust’s evolutionary origins and its biological function. In his book *Yuck* he outlines the complex nature of disgust stimuli and their ambiguous character (Kelly 2013). According to Kelly, disgust uses all of the human senses to detect so-called *universal disgust elicitors* to prevent contamination of the human body. Other disgust elicitors are taught and reinforced through individual and social learning mechanisms and vary depending on temporal, cultural, social and local factors. For example, consuming too much sugar is seen nowadays as a threat to one’s health and therefore potentially disgust eliciting. However, this has not always been the case and sugar has even been claimed to contribute to a healthy diet as shown in Fig. 2.

The detection of a disgust stimulus leads to heightened sensitivity for disgust elicitors as well as the increased capacity to memorize them, with the underlying incentive to prevent any kind of contact and consumption of entities that could harm the body (Kelly 2013). Kelly furthermore describes disgust as having a *downstream effect* by which disgust influences moral judgement, risk aversion, proprietary language and the display of disgust emotions including micro-expressions that—when perceived by others—work as a signaling system. What Kelly’s approach shows is that the unreflective perception of disgust elicitors is evolutionary developed, which contributes to the self-preservation of organisms. However, there are also many *phenotypic abnormalities* (e.g., being old, disfigured or fat) that can elicit disgust, without being an immediate threat to the organism’s self-preservation. This shows that disgust is subject to *social norms* (Ibid. 50). As we will explain in more detail below, current social norms that present being slender bodies as ideally healthy ones, help turn individuals affected by obesity into potential disgust elicitors.

## A phenomenological perspective on disgust

While Kelly’s theory is concerned with the biological function of disgust and its evolutionary origins, it (as Kelly admits) does not address the experience of disgust and how the disgusting entity^6^ is presented in it (Kelly 2013, 153). However, when concerned with understanding how disgust is embedded in public health campaigns, this experiential aspect of disgust must also be addressed to clarify *how* and *why* certain entities can be experienced as disgusting. One of the classical essays discussing the experience of disgust is Aurel Kolnai’s *On Disgust* first published in 1929. In this essay, Kolnai investigates from a phenomenological perspective how the disgusted subject apprehends the qualities of the entities it is disgusted by.^7^ His concern is thus not so much with whether these entities are inherently disgusting, but instead with how entities appear when they are apprehended as such. We will discuss his essay to outline how disgust can be understood as an affect that shapes how a particular entity is experienced. Thus, in contrast with Kelly’s account that seeks to unravel the functional mechanisms *underlying* the emotion of disgust, Kolnai is concerned with how the emotion of disgust manifests for the disgusted individual (Kolnai 2004, 5).

For Kolnai, disgust manifests as a defense reaction—or a mode of aversion—to the *proximity* of a given entity (Kolnai 2004, 40). He gives examples of several kinds of entities that might give rise to a disgust response, ranging from physical objects such as rotten meat and dirt to certain behavioral dispositions of human beings such as mendacity (Korsmeyer and Smith 2004, 25).^8^ Kolnai starts analyzing the distinctiveness of the experience of disgust by contrasting it with fear. According to Kolnai, the most crucial distinction between these two modes of aversion is: in the case of fear, the fearsome object is experienced as such through its relation to the subject’s survival (i.e., the apprehension of danger); while in the case of disgust, it is the qualitative features of the object itself that elicit disgust. As Kolnai puts it: “the intention of fear is […] principally directed towards *being*, towards the existential situation [*Daseinslage*] which confronts us and the intention of disgust towards features of the object, towards a type of *so-being* [*Soseinsart*]” (Kolnai 2004, 44).

Hence, in contrast with cases of fear, the experience of disgust is marked with an aversion from the object, but not in such a way that it immediately triggers a fight or flight survival mechanism. In itself, a disgusting object does not directly pose a threat to the existence of the subject, such that the emotion can in principle be eliminated quite easily by turning away one’s gaze, or moving away from the object (Ben-Ze’ev 2001, 389). However, there are some specific features to the experience of disgust that might hinder such easy aversions. Kolnai outlines several of these features: (1) the experienced proximity of the entity, (2) the low evaluation of the entity, and (3) how the entity attracts despite it being disgusting. Let us briefly discuss each of these by comparing them to the experience of fear:

1. In the experience of fear the proximity of the object is immediately negated by turning away from it (and hence removed from experience). However, the disgusting entity remains intact *through* its proximity; to be disgusted by something implies being disgusted by its perceived immediate proximity. In its proximity to the subject, the disgusting qualities of an entity stand out and remain intact, such that its disgustingness remains present until removed from proximity. This leads to the expectation that the disgusting entity itself either stops being disgusting, or removes itself from view without the subject’s intervention (Kolnai 2004, 41). A similar logic seems to be inscribed both into health campaigns explicitly using disgust, as well as into those focusing on “making healthy food the easy choice”: both presume that the entity that is associated with disgust should remove itself from proximity by starting to exercise sufficient self-control on their behavior and appearance itself (cf. Rich and Evans 2005).
2. In the case of fear, the fearsome object presents itself as being stronger than oneself, thereby necessitating the subject to flee in order to safeguard its existence. The experience of disgust, however, is characterized not by an activity of the subject, but instead by the desire that the object removes itself, and to stop disturbing the disgusted subject. Rather than the subject safeguarding itself, disgust makes it that its object is taken to be unworthy of existence, such that it must be cleansed from the subject’s surroundings (Kolnai 2004, 42). It is the object *as* an object presenting and spreading its disgustingness that it is considered to be *unworthy* of being present. Appealing to this disgust in the context of health promotion, then, if Kolnai is correct, involves simultaneously that certain entities must conform to a certain healthy ideal. This could potentially explain why people with obesity experience weight discrimination in professional contexts, leading to wage penalties and wrongful termination (Puhl and Heuer 2009).
3. Paradoxically, disgusting entities are often not straightforwardly averted by the subject, but also invite fascination; they have what can be called a “macabre allure” (Kolnai 2004, 42; Korsmeyer 2018). That is, the proximity of a certain entity that constitutes the experience of disgust involves the experience of being interested in the specific features of the entity and its reasons for being disgusting (Kolnai 2004, 43). In its proximity, the disgusting entity presents itself as striking, but also as veiling something, and inviting elaboration and analysis (Kolnai 2004, 47). This might explain why people are fascinated watching contestant with overweight attempting to lose weight as part of TV shows.

For the purposes of this paper, we refrain from discussing the variety of objects that might elicit disgust that Kolnai differentiates, but immediately turn to the matter relevant for our understanding of public health campaigns attempting to counter obesity: the relationship between disgust and the absence of self-control.^9^ For Kolnai, the absence of self-control signifies an absence of a more general plan or framework that people submit to, which he describes as an excessive vitality of life that is not structured by purpose, and is redundantly oscillating towards death (Kolnai 2004, 72). In such cases, Kolnai thus approaches self-control normatively: it functions as a way of imposing purpose on life, such that it can transcend its vital needs to follow a specific purpose. As a result, the disgust arising by witnessing an act that allegedly displays absence of self-control is not only directed at this act in isolation, but extends to the personality of the agent that is perceived as being unable to give purpose to her life (Kolnai 2004, 82–83).

## The performativity of disgust and the appeal to self-control in the “war against obesity”

If the above analysis is correct, then the appeal to self-control in anti-obesity campaigns focusing on a healthy lifestyle continues to present the overweight individual as a potential object of disgust. However, as Winfried Menninghaus notes when commenting on Kolnai’s work, the somewhat arbitrary way in which Kolnai integrates certain entities into his list of disgust elicitors is implicitly “shored up with an ideology of the “healthy” and “correct'' that itself goes unquestioned” (Menninghaus 2003, 19). This points to the fact that potentially any list of disgusting elicitors is not fixed, but rather dependent on certain ideological preferences and specific cultural and historical backgrounds. When recognizing that entities that elicit disgust are dependent on cultural and social norms, it becomes clear that framing the discourse around certain entities (e.g., high-calorie fast-food and its consumption) in a certain way actively transforms these as well as their consumers into potential disgust elicitors.

Kolnai’s phenomenological perspective does not address why certain objects come to be experienced as disgusting through socio-cultural norms and practices. Explaining this requires drawing attention to what Sara Ahmed calls the *performativity of disgust*: the processes through which objects are transformed by systematically framing them as being disgusting (Ahmed 2014, 85).^10^ For example, during the nineteenth century signs of overweight were being framed as indicators of a lack of control and indicators of an uncivilized society which established them as new disgust elicitors. This is further contributed to by the systematic framing of obesity as an indicator of disease (e.g., Ortiz et al. 2017).

On Ahmed’s account, disgusting objects are constituted as such—and this is how the performativity of disgust manifests—by attaching the affective response of disgust to a certain object and making it stick to it through the repetition in which its disgustingness is systematically emphasized (Ahmed 2014, 93). This “stickiness” of disgust remains underemphasized in Kolnai’s phenomenological approach, but can be observed in instances in which a certain entity becomes disgusting through its being in contact with another one. For example, when a breastfeeding mother eats unhealthy fast-food, she herself becomes a disgust elicitor in the context of feeding her child. The mother breaks with a moral norm and the consumption of a disgust elicitor (the burger in this case) has transformed her in a lasting way. Ahmed generalizes this idea when stating that the performativity of disgust consists in the repetition of a necessary bond between two entities, such that even when one of them is not visibly present, this bond remains to be experienced, thereby being constitutive of the disgust response to a given entity.

In what sense can the appeal to self-control in the “war against obesity” be said to take recourse to a similar performativity? When viewing disgust in terms of its performativity, it becomes clear that “being disgusting” is not an innate property of entities, but is the result of an entity being transformed into a disgusting one. As such, the individual that might exercise too little self-control is not a potential disgust elicitor on its own, but only because (a) the absence of self-control is already perceived as disgusting; and (b) because the absence of self-control is a character trait consistently connected to overweight. As we have established earlier in this paper, both the condemnation of excess and the link between excessive food intake and being fat are not unique to the discourse around obesity that arose in the last couple of decades, but have deep historical roots. Furthermore, and this seems to be a phenomenon more particular to the discourse around the “war against obesity”, self-control is linked not only to a particular (visible) instance of excessive food intake, but the lifestyle of an individual with overweight as a whole is transformed into something that can be condemned and elicits disgust accordingly.

By continuing to present overweight as intimately connected to self-control, advertisements and campaigns that draw attention to the benefits of living a healthy lifestyle can be considered to be what Ahmed understands as processes within which the performativity of disgust manifests. They do so through a repetition in which self-control and a healthy lifestyle are linked together, thereby presenting other lifestyles as being the result of the negation of self-control. As a result, lifestyles deviating from the healthy standard are presented as something that one must distance oneself from. Through the cultivation of one’s own self-control one becomes present as a subject for which certain excesses appear as disgusting and to be avoided.

**Fig. 3:**
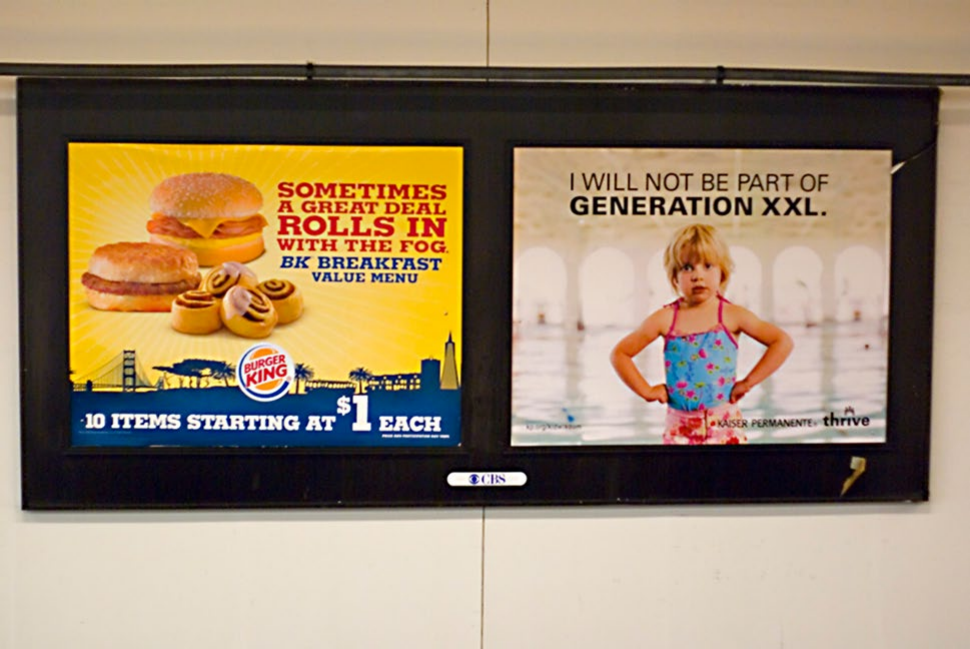
The image shows two different billboards. One advertises fast food and the second is a health campaign showing a child with the headline “I will not be part of generation XXL”. Photo: “Hypocrisy” by Sathish (2007) is licensed under the Creative Commons license CC BY-NC-ND 2.0

An example of this performativity of disgust can be seen in Fig. 3. We see on the left side an advertisement for a particular breakfast at a fast food restaurant, with an anti-obesity advertisement next to it, where one sees a child stating that it “will not be part of generation XXL”.^11^ The intended effect of the poster on the right side is based on the perceived link between the consumption of particular food items and the development of a particular body (one that can apparently be characterized as XXL). The poster on the left is a campaign promoting fast-food products. However, due to the performativity of disgust, the products in this context can be interpreted as disgust elicitors representing a lack of control of people with obesity. Such a campaign might be less stigmatizing than showing people with increased body weight, but such an approach is likely to reinforce established moral concepts of the “right” kind of food and “correct” way of eating. This, again, shows how the disgust elicited by the consumption of particular food items remains to stick to the body and character of the individual engaging in the act of consumption (Fig. 1).

## Identity and the morality of food

Food often contains moral meaning, and while pleasure experienced when eating food is not seen as disgusting as such, it is expected from the individual to show restraint and moderation by avoiding deliberate food excess (Askegaard et al. 2014). One of the main reasons for this expectation is, as Quill Kukla has recently argued, that food and food consumption are intimately tied up with one’s social identity (2018). Based on her argument, we briefly suggest in this section that the close connection between food and social identity shows that the consumption of certain items and amounts of food and engaging in certain lifestyle choices serve to continuously reaffirm one’s identity, and make it that other food consumption and lifestyle choices are linked to other identities that are rejected. For example, following a vegan diet can closely be linked to one’s self-perception and way of living. From this perspective, every other food identity might be one of excess, leading to the condemnation and stigmatization of the purportedly excessive behavior.

What is crucial in Kukla’s argument is that the process through which other food consumption patterns are rejected is not unique to people striving to live a healthy (or vegan) lifestyle vis-a-vis other food identities, but is part of every food identity. The current discourse around obesity and promoting healthy food choices is often based on the assumption that so-called “unhealthy food” is free of pleasure. For example, by assuming that eating a burger at a fast-food restaurant is actually a very poor experience in terms of its taste. However, often the contrary seems to be the case in practice: we associate healthy food as requiring controlled eating and to be “cold and unerotic” (Kukla 2018, 599). It therefore seems that the promotion of a “healthy” food item is based on the basic premise that it is the right thing to eat rather than the most flavorsome one.

**Fig. 4:**
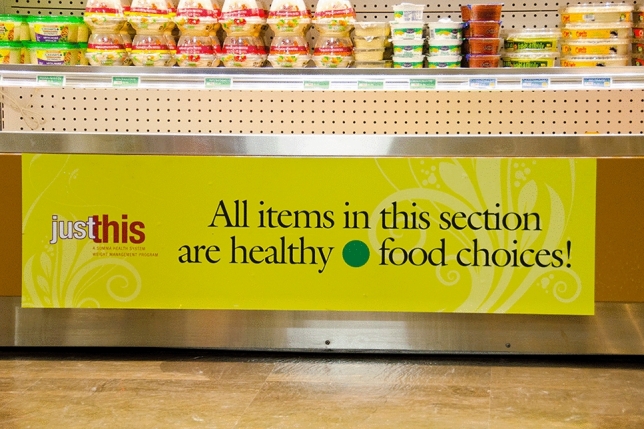
The image shows a cafeteria in a hospital environment that offers healthy food choices to patients and staff. Nudging consumers to make healthy food choices has proven to be quite challenging. When consumers have to choose between healthy (associated with lacking taste) and unhealthy food (associated to be tasteful) items, taste often prevails. Photo: “Summa Barberton Hospital Cafeteria Grand Opening” by Barberton Community Foundation (2012) is licensed under the Creative Commons license CC BY-NC-ND 2.0

Consider for example Fig. 4 which shows the cafeteria in a hospital. Food items have been selected for staff and patients based on their perceived health benefits rather than flavor profile. This might be beneficial in a hospital environment but could be controversial in a standard supermarket. The perceived degree of “unhealthiness” of a food item ironically enhances its attractiveness and perceived level of flavor (Raghunathan et al. 2006; Mai and Hoffmann 2015). Choosing a food item based on the associated health benefit, therefore, relies on making the “right choice” which again is based on the concept of being in control. When Kukla is correct, a focus on lifestyle choices in public health campaigns seem to always trigger negative emotions towards other food identities (such as disgust). Hence, the focus on making specific lifestyle choices over others therefore similarly gives rise to feelings of disgust towards certain groups and behavior patterns, just as the explicit presentation of (the interior of) overweight bodies and the eating practices of individuals with overweight would do.

## Closing remarks

In this paper, we described the different ways in which disgust can manifest in the context of anti-obesity campaigns. We highlighted that there might be good reasons to think that a focus on self-control in health campaigns and advertisements tacitly reintroduces an image of people affected by obesity as being disgusting—albeit in another manner. In doing so, our intention is not to condemn the focus of advertisements on “making the healthy choice the easy choice” but rather to point out that the emotion of disgust is deeply embedded in our perception of the “right way to eat” the “right food,” and has long-standing historical roots.

Our discussion of how entities are constituted as disgusting and experienced as such in a variety of ways reveals that being disgusted by a body with overweight is not necessarily the result of being confronted with its physical traits. Rather, the performativity of disgust makes it that entities are experienced as disgusting due to them being connected to certain actions (eating specific amounts at a specific pace), objects (eating specific food such as with a high fat and sugar content), moral judgments (refusing to eat healthy food), or character traits (having no willpower). Furthermore, the entanglement of the food choices one makes (and the food choices that one explicitly rejects) and one’s identity indicates that the experience of disgust involves a rejection of the individual that makes a choice that is perceived as “wrong” or “deviant” (see Fig. 1). In other words, disgust is not only elicited through the presentation of an entity with certain features but can also be elicited through the subject’s (perceived) unwillingness or inability to accept the “easy and healthy food offer”.

Instead of presenting certain food items as disgusting, these campaigns emphasize the positive qualities of them, thereby presenting their consumption as helping to realize a healthier and more pleasurable lifestyle. However, as our analysis of the experience of disgust and the performative constitution of disgusting objects shows, it seems unlikely that food items can be isolated from the subject consuming them. That is, the choice for healthy food remains to be portrayed as one that can only be made when the subject has sufficient self-control and moderation that allows the consumption of the “right” amount of the “right” food items (i.e., the items presented as healthy). As a result, the promotion of certain food items as being the healthy ones (and other ones as disgusting) remains to suggest that one’s (potentially disgusting) body size is the consequence of one’s (potentially disgusting) character traits. Insofar as campaigns against obesity should not lead to the stigmatization of individuals affected by obesity and the spread of a negative public attitude towards people affected by obesity by forging a connection between overweight and the absence of self-control, it is crucial to recognize that they potentially remain ingrained in a pedagogy of disgust. Doing so requires (1) to acknowledge that promoting the consumption of certain food items amounts to the promotion of a certain identity that involves certain norms about which behaviors are appropriate and inappropriate; and (2) that the promotion of a certain choice as being the healthy one simultaneously reinforces an image in which the subject engaging in other actions can be held responsible in terms of certain behavioral dispositions such as self-control and the absence thereof.

## Footnotes

1 We refer to the term “war against obesity” in quotation marks since the term employs a war-like metaphor. Comparing the fight against obesity with a war-like situation seems to imply that all means to reduce the condition's prevalence can be justified (e.g., Barry et al. 2009; Sontag 1978).

2 We will refer to the term "obesity epidemic" in quotation marks because the condition does not meet the definition of disease. It is furthermore not clear if increased body weight itself is a health risk as many people with overweight suffer no impairment as a consequence of their increased weight (Heshka and Allison 2001).

3 However, it needs to be pointed out that even though excessive eating and signs of fatness have been criticized throughout the centuries by some scholars and medical professionals, there were also periods during which an increased body weight represented prosperity and wealth (Forth 2012a, b, 2013).

4 Devisch and Vanheule (2015) draw from Foucault to show how modern governments are increasingly concerned with governing the biological aspects of life on the basis of particular norms about what is a healthy population. They analyse the promotion of a healthy lifestyle through the framework of governmentality (e.g., Foucault 2007), and suggest that the discourse around healthy lifestyles is one in which a healthy lifestyle is something that citizens should choose freely and autonomously, while it simultaneously limits this freedom by maintaining that the particular norms of health professionals should be followed to make “correct” choices (Devisch and Vanheule 2015, 430). Although we acknowledge this process, in this paper we are primarily concerned with how this very discourse constitutes individuals affected by obesity can be *experienced* as disgust eliciting. As a result, we approach the topic of obesity primarily through a phenomenological lens. A critical analysis of how a Foucauldian and a phenomenological perspective can complement one another is beyond the scope of this paper.

5 It is sometimes argued that putting the blame of overweight on individuals that allegedly lack the capacity to avoid risks and exercise sufficient self-control is part of a broader individualistic neoliberal discourse (Guthman and DuPuis 2006; LeBesco 2011). A detailed discussion of this issue is beyond the scope of this paper, as our primary concern is with the different ways in which individuals with overweight can become present as potentially disgust eliciting.

6 We use the term “entity” to capture both that objects (e.g., feces) and humans (e.g., individuals with a certain character or certain physical traits) can be apprehended as disgusting, without making a prior distinction of how those different entities are experienced in disgust.

7 In this paper, we focus on how Kolnai’s phenomenological approach helps explaining the relation between disgust and the absence of self-control. It is beyond the scope of this paper to firmly embed Kolnai’s thought into the phenomenological tradition of thinking about the body. For an excellent discussion of Kolnai’s relation to Husserlian phenomenology, see Korsmeyer and Smith (2004).

8 Kolnai gives several examples of his personal experiences with disgust of which it would be currently controversial to be discussed on these terms, such as male homosexual advances (Korsmeyer and Smith 2004, 25). We reject the idea that a final list of disgust elicitors independent of an individual, social, cultural or historical context can be produced, and take such examples to be indicative of Kolnai’s individual preferences and the historical period in which the essay was written. However, we believe that his phenomenological analysis of disgust *as* a mode of aversion remains of value when analyzing the experience of disgust—regardless of what this disgust is elicited by.

9 Note that by making this connection, we do not in any way presuppose that there *should* exist a relation between disgust and the absence of self-control. Rather, we intend to propose a way of understanding the ongoing stigmatization of individuals affected by obesity (Ringel and Ditto 2019) by revealing how current public health campaigns—in spite of best intentions—might implicitly take recourse to the pedagogy of disgust they intend to depart from.

10 When turning to the performativity of disgust, we surpass the question as to whether there are indeed entities that are *essentially* disgusting (e.g., feces), but instead point to the fact that many entities elicit disgust not due to their innate properties, but through the way in which they are *made* disgusting in particular sociocultural contexts.

11 This example nicely shows that the development of specific healthcare campaigns can be understood as a reaction of the extensive advertising of junkfood in many societies.
